# Longitudinal changes in DNA methylation during the onset of islet autoimmunity differentiate between reversion versus progression of islet autoimmunity

**DOI:** 10.3389/fimmu.2024.1345494

**Published:** 2024-06-10

**Authors:** Patrick M. Carry, Lauren A. Vanderlinden, Randi K. Johnson, Teresa Buckner, Andrea K. Steck, Katerina Kechris, Ivana V. Yang, Tasha E. Fingerlin, Oliver Fiehn, Marian Rewers, Jill M. Norris

**Affiliations:** ^1^ Colorado Program for Musculoskeletal Research, Department of Orthopedics, University of Colorado, Aurora, CO, United States; ^2^ Department of Epidemiology, Colorado School of Public Health, Aurora, CO, United States; ^3^ Department of Biomedical Informatics, School of Medicine, University of Colorado, Aurora, CO, United States; ^4^ Department of Kinesiology, Nutrition, and Dietetics, University of Northern Colorado, Greeley, CO, United States; ^5^ Barbara Davis Center, Department of Pediatrics, University of Colorado, Aurora, CO, United States; ^6^ Department of Biostatistics and Informatics, Colorado School of Public Health, Aurora, CO, United States; ^7^ Department of Medicine, University of Colorado, Aurora, CO, United States; ^8^ Department of Immunology and Genomic Medicine, National Jewish Health, Aurora, CO, United States; ^9^ University of California Davis West Coast Metabolomics Center, Davis, CA, United States

**Keywords:** DNA methylation, type 1 diabetes (T1D), DAISY, islet autoimmunity, reversion

## Abstract

**Background:**

Type 1 diabetes (T1D) is preceded by a heterogenous pre-clinical phase, islet autoimmunity (IA). We aimed to identify pre vs. post-IA seroconversion (SV) changes in DNAm that differed across three IA progression phenotypes, those who lose autoantibodies (reverters), progress to clinical T1D (progressors), or maintain autoantibody levels (maintainers).

**Methods:**

This epigenome-wide association study (EWAS) included longitudinal DNAm measurements in blood (Illumina 450K and EPIC) from participants in Diabetes Autoimmunity Study in the Young (DAISY) who developed IA, one or more islet autoantibodies on at least two consecutive visits. We compared *reverters* - individuals who sero-reverted, negative for all autoantibodies on at least two consecutive visits and did not develop T1D (n=41); *maintainers -* continued to test positive for autoantibodies but did not develop T1D (n=60); *progressors -* developed clinical T1D (n=42). DNAm data were measured before (pre-SV visit) and after IA (post-SV visit). Linear mixed models were used to test for differences in pre- vs post-SV changes in DNAm across the three groups. Linear mixed models were also used to test for group differences in average DNAm. Cell proportions, age, and sex were adjusted for in all models. Median follow-up across all participants was 15.5 yrs. (interquartile range (IQR): 10.8-18.7).

**Results:**

The median age at the pre-SV visit was 2.2 yrs. (IQR: 0.8-5.3) in progressors, compared to 6.0 yrs. (IQR: 1.3-8.4) in reverters, and 5.7 yrs. (IQR: 1.4-9.7) in maintainers. Median time between the visits was similar in reverters 1.4 yrs. (IQR: 1-1.9), maintainers 1.3 yrs. (IQR: 1.0-2.0), and progressors 1.8 yrs. (IQR: 1.0-2.0). Changes in DNAm, pre- vs post-SV, differed across the groups at one site (cg16066195) and 11 regions. Average DNAm (mean of pre- and post-SV) differed across 22 regions.

**Conclusion:**

Differentially changing DNAm regions were located in genomic areas related to beta cell function, immune cell differentiation, and immune cell function.

## 1 Introduction

T1D is an autoimmune disorder with significant long-term morbidity. The pre-clinical phase is defined by the appearance of autoantibodies against pancreas cell antigens, termed islet autoimmunity (IA). There is strong evidence to support autoantibodies as a biomarker of T1D risk (1). However, IA is dynamic. While progression to T1D or multiple autoantibodies has been well characterized, a subset of individuals lose autoantibody positivity (2) and revert back to an autoantibody negative state. Autoantibody reversion was first described by Spencer et al (3) in a cohort of 685 individuals with a first degree relative affected by T1D. After 5 years, 7/20 developed T1D, 1 remained AB positive and 12/20 reverted. Transient autoantibody positivity has been described in several additional studies (4–6). However, these historical studies describing the transient nature of autoantibodies are difficult to interpret due to the development of more accurate autoantibody tests as well as differences in the definition of reversion. Vehik et al (2) conducted the most comprehensive and rigorous study of reversion in current literature. Among 596 individuals enrolled in The Environmental Determinants of Diabetes in the Young (TEDDY) study who developed one or more persistent autoantibodies, 21% reverted to an antibody negative state. Seroreversion was associated with significantly decreased risk of T1D (hazard ratio: 0.14, 95% CI: 0.04-0.59). Understanding the unique protective mechanisms occurring prior to or following IA that lead to IA reversion may have important implications for development of interventions that delay or prevent progression to T1D.

Genetic variation is a well-established risk factor for T1D (7). However, heterogeneity in disease concordance among monozygotic twins (8) as well as temporal changes in both T1D incidence (9) and age at T1D onset (10) in population studies have created a strong interest in the role of the environment in the etiology of T1D. Epigenetic modifications such as DNA methylation (DNAm) may represent a mechanistic pathway between genetic susceptibility, environmental exposures, and progression or reversion of IA. Epigenetics broadly describes a class of modifiable mechanisms that can regulate gene expression and are sensitive to external stimuli (11). DNAm is a frequently studied epigenetic biomarker that is postulated to play a role in autoimmune diseases as epigenetic mechanisms are important regulators of immune cell differentiation, plasticity and function (12, 13). DNAm changes prior to and during the IA phase may provide key information about underlying epigenetic profiles that explain progression or reversion from IA.

Previous epigenome wide studies have identified significant associations between DNAm and T1D (14–17). However, associations have been inconsistent and many of the studies have focused on static and/or post-T1D differences in DNAm between cases and controls (14–16). Although important in understanding the etiology of T1D, DNAm differences obtained from a single time point are difficult to interpret as it is not possible to determine when the changes occurred and moreover, whether they are the cause or consequence of the disease process. Understanding the timing of the changes is key to identifying external factors that cause these changes and therefore, may be amenable to preventative interventions. The purpose of this study was to test DNAm obtained before and after IA seroconversion (SV) in the Diabetes Autoimmunity Study in the Young (DAISY). We aimed to identify pre vs. post-SV changes in DNAm that differed across three distinct IA progression phenotypes, those who lose autoantibodies (reverters), progress to clinical T1D (progressors), or maintain autoantibody levels (maintainers).

## 2 Materials and methods

### 2.1 Study population

We reviewed individuals from the Diabetes Autoimmunity Study in the Young (DAISY) who developed islet autoimmunity (IA) between February 1994 and February 2019. DAISY is a longitudinal birth cohort study that includes n=2544 children at high risk for T1D. Subjects are recruited from two high risk populations, those with a first degree relative (FDR) with T1D or those with a high-risk genotype, [defined as DRB1*04, DQB1*0302/DRB1*0301, DQB1*0201 (DR3/4 DQ8)]. Subjects complete study visits at 9, 15, and 24 months. Following the 24-month visit, study visits occur annually. As described previously (18), radio-immunoassays were used to test serum samples for autoantibodies to insulin (IAA), GAD65 (GAA), and IA-2 (IA-2A). Prior to 2010, GADA and IA-2A were tested using a combined radioassay (19). The National Institute of Diabetes and Digestive and Kidney Diseases harmonized assay was used to test for GADA and IA-2A after 2010 (20). Serum samples from individuals positive for GAD65, IAA, or IA-2 were tested for ZnT8A following development and implementation of the ZnT8 assay (21). If autoantibodies are detected, participants return for study visits every 3-6 months.

Islet autoimmunity (IA) was defined as the presence of one or more autoantibodies (see above) on at least two consecutive visits 3-6 months apart. The first visit among these consecutive autoantibody positive visits designated the start of IA, referred to as seroconversion (SV) throughout the remainder of the manuscript. We defined the three autoimmune progression phenotypes based on the autoantibody testing. The *reverter group* was defined as individuals who reverted for all autoantibodies during two or more consecutive visits, did not develop T1D, and were autoantibody negative for all autoantibodies at their last DAISY visit. The *maintainer group* was defined as individuals who continued to test positive for islet autoantibodies and did not develop T1D at the time of their last visit. The *progressor group* was defined as individuals who developed clinical T1D.

Among individuals who developed IA during DAISY and underwent autoantibody testing for a minimum of two or more study visits (n=213), we excluded individuals for the following: missing a pre- or post-SV blood sample (n=54), onset of IA unclear due to gaps (>365 days) in study visits (n=2), missing study visit prior to initial pre-SV positive visit (n=14). The Colorado Multiple Institutional Review Board approved all DAISY protocols (COMIRB 92-080). Informed consent and assent, if appropriate, was obtained from the parents/legal guardians of all children prior to participation in any research related activities.

### 2.2 Methylation measurements

Methylation measurements were obtained from peripheral whole blood samples collected at multiple time-points in individuals from DAISY. The Infinium HumanMethylation 450K Beadchip platform (Illumina, San Diego, CA, USA) was used to obtain methylation measurements on a subset of samples. The 850K Infininium MethylationEPIC BeadChip (Illumina, San Diego, CA, USA) was used to obtain measurements on the remaining samples. Two platforms were used due to changes in technology during the course of the study. Samples were randomly assigned to the two platforms making sure all timepoints from the same individual were included on the same platform.

DNA was bisulfite converted using the Zymo EZ DNA Methylation kit (Zymo Research, CA, USA). The bisulfite-converted DNA was labeled with fluorescent dyes and hybridized to 450K and 850K DNAm arrays. Samples were arranged on the plates in a specific sequence to minimize within and between batch effects (plate effects are represented by first 11 digits of the array variable on GEO). The minfi (v1.12.0) package (22) in R (v3.5.2) was used to perform quality control (QC) checks at the sample level. The processing pipeline is described in greater detail in Vanderlinden et al (23).

**Figure 1:**
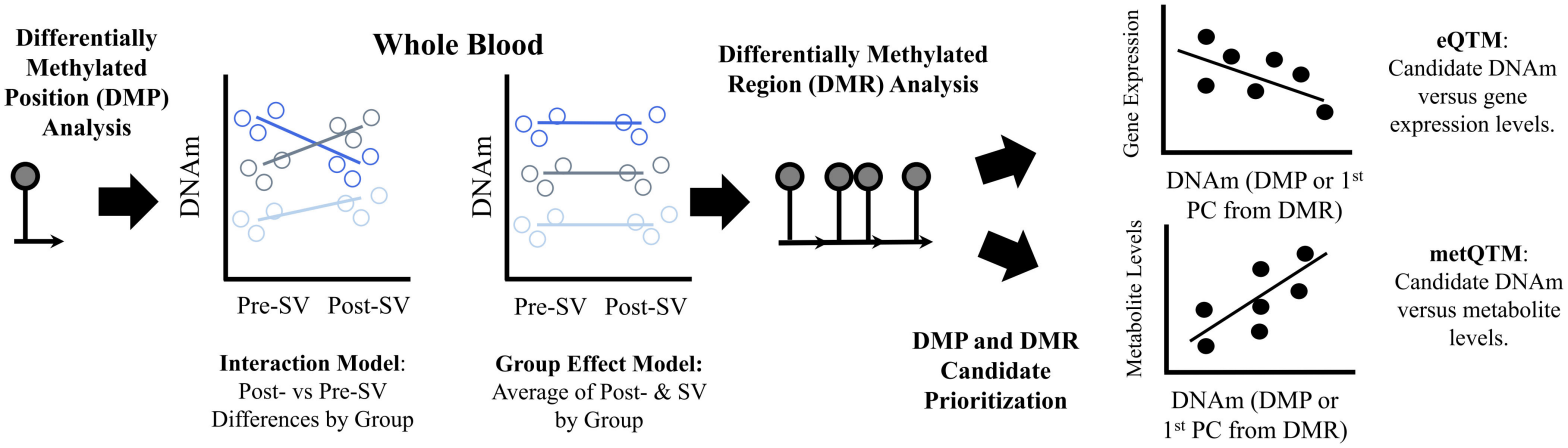
Summary of methods used to identify and prioritize DNAm candidates. Description: We used an epigenome wide association study design to identify differentially methylated positions (DMP) associated with the three islet autoimmunity progression phenotypes, reverters, maintainer, or progressors. We used two DMP models (1) an interaction model that tested whether changes in DNA methylation (DNAm) levels at single CpGs pre-IA versus post-IA differed across groups and (2) a group effect model that tested whether average methylation levels (pre- and post-IA) differed across groups. We also performed regional analyses (differentially methylated regions or DMRs) based on single CpG sites from the two models to identify regions with consistent methylation effects. We identified regions where average regional methylation levels differed between groups (μDMRs) as well as regions where changes in regional methylation levels pre- vs post-IA differed across groups (ΔDMRs). In order to prioritize regions, we tested whether the DNAm candidates identified in our analysis were associated with gene expression levels post-SV, an expression quantitative trait methylation analysis (eQTM). To account for the multiple CpGs within each DMR, we used a principal component analysis to capture common patterns across all CpGs included in the DMR. We identified cis-eQTMs (midpoint of region +/- 500 KB of the TSS of the gene) by testing the correlation between gene expression and the 1st principal component. We also tested the correlation between DNAm candidates and metabolite levels obtained from overlapping samples, a metabolite quantitative trait methylation analysis (metQTM). We used a principal component analysis to capture common patterns across all CpGs included in the candidate DMRs. We tested the correlation between metabolite levels and the 1st principal component. CpGs are represented by lollipop plots in the figure.

The DNAm probes were annotated to the genome based on the hg19 genome build using the Illumina annotation manifest files. Non-autosomal CpGs or CpGs located within or near (<2 base pairs) known single nucleotide polymorphisms (SNPs) were excluded. CpG sites with a beta range <3% on both platforms were removed from analysis. A total of n=198,008 overlapping DNAm probes met our filtering criteria and were used in subsequent analyses. Normalized M-values (SeSAMe (v1.0.0) pipeline with Noob normalization) were used in all statistical analyses. We use the term DNAm probe and the probe identifier when referring to the data in the Methods and Results. However, each probe is designed to measure DNAm at a single CpG site which is used as a more general term in the Discussion. See **Figure 1** for an overview of the study methods.

### 2.3 Overlapping gene expression measurements

Gene expression data were available in a subset of individuals (n=36) at the post-SV visit. RNA processing and quantification is described in greater detail in Carry et al (24). In brief, paired end sequencing was performed using the Illumina NovaSEQ 6000™ system and samples were quantified against the Ensembl reference transcriptome (hg19, version 87) using the RSEM algorithm (25). Data were quantile normalized using DESeq2 (26), re-normalized using RUV (27), and then transformed using the regularized log function (26). The transformed data were used in all subsequent statistical analyses.

### 2.4 Overlapping metabolomics measurements

Untargeted metabolomics data were available in a subset (n=110) of individuals at both the pre-SV and post-SV visits. Metabolomics processing and quantification is described in greater detail in Carry et al (28). In brief, non-fasting plasma samples were used to quantify metabolite levels using three untargeted panels, HILIC panel: HILIC-QTOF MS/MS (29), GCTOF panel: GC-TOF-MS (30), and Lipid panel: CSH-QTOF MS/MS (31). BinBase (32) was used to process and annotate the GC-TOF-MS data. MS-Dial (33) was used to process and annotate the liquid chromatography (LC), CSH-QTOF-MS and HILIC-QTOF-MS, data. LipidBlast (34) and Massbank of North America were also used to annotate the complex lipids (http://mona.fiehnlab.ucdavis.edu/). Metabolomic data were normalized using the systematic error removal using random forest (SERRF) algorithm (35). All metabolites were Box-Cox transformed prior to statistical analysis.

### 2.5 Genetic ancestry

Ancestry principal components (PC) were estimated for all study participants from genetic data collected in DAISY. Sample processing and genotyping were performed at the University of Virginia School of Medicine Center for Public Health Genomics based on exome genotyping (Illumina HumanCoreExome-24 BeadChip, N=283) or whole genome sequencing (N=162) from the larger DAISY population, see Buckner et al (36) for a more complete description of the genetic processing and calculation of the genetic ancestry PCs.

### 2.6 Statistical analyses

The overall methods workflow is summarized in **Figure 1**. Linear mixed models were used to test for differences in DNAm between the pre- and post-SV visit across reverters, maintainers, and progressors (autoimmune phenotype*visit interaction). Separate linear mixed models were also used to test for differences in average DNAm (mean of the DNAm levels at the pre- and post- SV visits) between the autoimmune phenotypes (group effect). Platform (EPIC vs 450K), age, sex, and cell proportions (estimated using the minfi (v1.12.0) package (22) implementation of the Houseman method) were adjusted for in all models. The group effect models were also adjusted for population ancestry (see **Supplementary Material** for complete description of ancestry data). Ancestry data (1^st^ 2 PCs) were unavailable for 2 individuals in the group effect model and thus, these individuals were not included in this analysis. See **Appendix 1 (Data Sheet 1)** for the linear mixed model code. We did not adjust for ancestry in the interaction (autoimmune phenotype*visit) models because the interaction models test for within individual differences, and thus are less likely to be impacted by time invariant confounders such as population ancestry. The Benjamini Hochberg false discovery rate (FDR), was used to correct for multiple comparisons (37). Significance was assessed based on the FDR adjusted p-value <0.10. Model diagnostics are described in the **Supplementary Files (Data Sheet 2)**, see **Appendix 2, Figures A–C** and **Table A**.

The comb-p python software package (38) was used to identify differentially methylated regions (DMRs). Within the comb-p pipeline, we used a seed p-value of 0.1 and then searched for adjacent probes within a window of 500 bases, using a step size of 50 bases. Comb-p combines probes within this window and then calculates an overall, spatially corrected p value for the entire region based on the Stouffer-Liptak method. The Sidak method is used to adjust the overall regional p values for multiple testing. Regional analyses were performed based on the individual DNAm probes from the interaction (post- vs pre-SV changes by autoimmune phenotype), referred to as differentially changing DMRs (ΔDMR) throughout the remainder of the manuscript. Regional analyses were also performed based on DNAm probes from the main effect model (differences in average of pre- and post-SV DNAm between groups), referred to as average DMRs (μDMR) throughout the remainder of the manuscript. For both regional analyses, we reviewed all regions with ≥4 DNAm probes that were significant at the combined Sidak adjusted region p value of 0.10. Because the interaction and group effect p values are based on a two degree of freedom test (numerator degrees of freedom for the overall F-test), it is possible for the DMR to capture a set of DNAm probes with similar p values but substantial heterogeneity in the directions of effect within the three groups. Therefore, for the ΔDMRs, we retained regions with a consistent direction of effect, defined as a region where the direction of change in DNAm between the two visits (hyper methylation or hypo methylation) was consistent across 100% of the DNAm probes within the region in one or more of the study groups. For the μDMRs, we retained regions where the direction of effect (hypo or hypermethylation) for one or more of the pairwise group comparisons was consistent across 100% of the DNAm probes included in the region.

### 2.7 Expression quantitative trait methylation analysis: correlation between gene expression and DNAm candidates

In order to better understand our primary DNAm results, we tested the correlation between gene expression levels and our DNAm candidates, one DMP, 11 ΔDMRs, and 22 μDMRs in a subset of individuals (n=36, see **Appendix 3, Table B**) with methylation data pre- and post-SV as well as gene expression data post-SV. First, linear mixed models were used to regress out age, sex, platform, and cell proportions from the DNAm values at each of the candidate CpG sites. Ancestry PC1 and ancestry PC2 were also regressed out from all CpG sites included in the μDMRs candidate regions. Next, using the residuals from the linear mixed models, the within individual differences in DNAm (post-SV minus pre-SV) were used to represent changes in DNAm between the study visits for each of the CpG sites included in the ΔDMRs. The average residual values from the post-SV and pre-SV study visits were used to represent average methylation for each of the CpG sites within the μDMRs. Next, we performed a principal component analysis of DNAm levels across the region-specific CpG sets. For each DMR, the first PC was extracted for subsequent testing, allowing us to consider all CpG sites together rather than testing many individual sites separately. Linear regression models were then used to regress out the effects of age and sex from the gene expression levels. Finally, Spearman correlation coefficients were used to test the correlation between DNAm and gene expression residuals. We looked for cis-eQTMs, defined as genes significant at the FDR adjusted p value of 0.10 where transcription start site was +/- 500 KB of the midpoint of the DMR. FDR adjustment was based on the total number of DNAm cis-gene pairs (256 transcript DNAm pairs for the ΔDMR candidates and 544 transcript DNAm pairs for the μDMR candidates).

### 2.8 Metabolite quantitative trait methylation analysis: correlation between metabolite levels and DNAm candidates

We tested the correlation between DNAm and untargeted metabolite levels in a subset of our study population (n=110, see **Appendix 3, Table B**) with DNAm and metabolomics data available both pre- and post-SV. Only data from overlapping samples was included in this supplementary analysis. Linear models were used to regress age and sex from the Box-Cox transformed metabolite levels at each visit. Consistent with the DNAm methods, using the residuals from the linear mixed models, the difference between metabolite residuals at each visit (post-SV minus pre-SV residuals) was used to represent change in metabolites and the average residual values (average of post-SV and pre-SV residuals) were used to represent average metabolite values. For the ΔDMR candidates and the single DMP candidate, linear regression models were then used to test the correlation between the change in metabolites versus the ΔDMR PCs (described above) as well as the single DMP candidate. For the μDMR candidates, linear regression models were then used to test the correlation between average metabolite levels versus the μDMR PCs (described above). False discovery (FDR) rate adjusted p values were calculated for all individual metabolite DNAm candidate pairs according to methods described by Benjamini and Hochberg (37). FDR adjusted p values were calculated separately for each platform. Only annotated metabolites from the HILIC (81 metabolites), Lipid (373 metabolites), and GC-TOF (98 metabolites) panels were evaluated in subsequent analyses. Metabolites were evaluated at an FDR adjusted p value of 0.10.

## 3 Results

### 3.1 Study population

The final study population included 60 individuals in the maintainer group, 42 individuals in the progressor group, and 41 individuals in the reverter group. At both the pre-SV and post-SV visits, age differed by group, and the estimated cell proportions differed by group at the post-SV visit (**Table 1**). At the time of data analysis, duration of follow-up, defined as median time from the initial visit to the development of T1D or last study visit, was 9.3 years (IQR: 6.1 to 12.3 years) for the progressors, 16.5 years for the maintainers (IQR: 14.3 to 20.9 years) and 16.6 years for the reverters (IQR: 15.2 to 20.2 years).

**Table 1:** Demographics and clinical characteristics.

	Maintainer n=60		Progressor n=42		Reverter n=41		P Value
	Median | Freq	IQR | %	Median | Freq	IQR | %	Median | Freq	IQR | %	
Pre-Islet Autoimmunity Visit							
Age at Visit, median (IQR)	5.7	1.4-9.7	2.2	0.8-5.3	6.0	1.3-8.4	0.0079
CD8T, median (IQR)	13.3%	9.4-16.6%	14.6%	11.8-15.9%	12.2%	9.7-16.1%	0.3864
CD4T, median (IQR)	22.0%	15.6-26.1%	23.4%	17.3-31.8%	19.3%	16.1-25.5%	0.1959
NK, median (IQR)	1.4%	0.0-4.7%	0.0%	0.0-1.5%	1.3%	0.0-3.1%	0.0653
Bcell, median (IQR)	15.3%	10.6-18.5%	17.9%	13.4-22.6%	14.9%	10.2-19.7%	0.1599
Mono, median (IQR)	8.3%	6.9-10.3%	7.5%	5.2-9.4%	7.6%	6.2-9.5%	0.3390
Gran, median (IQR)	38.5%	30.6-50.9%	35.5%	24.7-44.6%	42.8%	32.0-52.0%	0.2205
Post-Islet Autoimmunity Visit							
Age at Visit, median (IQR)	8.0	5.2-11.3	4.9	2.4-9.4	7.1	3.1-10.0	0.0087
CD8T, median (IQR)	11.8%	9.5-15.6%	14.6%	11.3-16.7%	12.3%	8.9-16.4%	0.1183
CD4T, median (IQR)	17.6%	13.1-22.1%	21.7%	17.3-26.9%	17.6%	13.0-21.7%	0.0061
NK, median (IQR)	2.7%	0.0-6.0%	0.0%	0.0-3.5%	1.3%	0.0-4.1%	0.0018
Bcell, median (IQR)	11.2%	8.6-15.0%	16.5%	12.7-19.7%	13.1%	8.3-16.7%	0.0011
Mono, median (IQR)	9.1%	7.8-10.8%	7.8%	4.8-9.3%	8.5%	7.0-10.1%	0.0293
Gran, median (IQR)	46.0%	39.6-52.7%	37.9%	28.6-44.4%	47.8%	38.4-53.6%	0.0025
**Non-Hispanic White Ethnicity, freq (%)**	43	71.7%	38	90.5%	29	70.7%	0.0458
**Female Sex, freq (%)**	34	56.7%	19	45.2%	21	51.2%	0.5224
**HLDR3/4 High Risk Genotype, freq (%)**	16	26.7%	19	45.2%	10	24.4%	0.0711
**First Degree Relative with T1D, freq (%)**	38	63.3%	25	59.5%	19	46.3%	0.2242

IQR, interquartile range; CD8T, cytotoxic T cells; CD4T, T helper cells; NK, natural killer T cells; Mono, monocytes; Gran, granulocytes.

The specific autoantibody subgroups present at the onset of seroconversion in the three groups are described in greater detail in **Appendix 4 (Data Sheet 4), Table C**. As expected, the prevalence of multiple autoantibodies at serconversion was higher in progressors (31%) relative to maintainers (18%) and reverters (0%). Across the entire islet autoimmunity follow-up period, the occurrence of multiple autoantibodies at one or more study visit(s) following IA seroconversion was also higher in progressors (86%) compared to maintainers (58%). Among reverters, 10% developed multiple autoantibodies at one of more study visit(s) during the time period between seroconversion (IA onset) and seroreversion.

### 3.2 Differentially methylated position analysis

**Figure 2:**
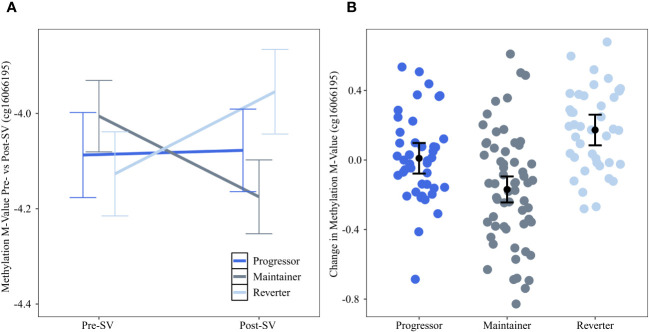
Changes in DNAm between the pre- and post-SV visits at cg16066195 across the three IA progression phenotypes. Description: **(A)** provides the average methylation M-values and corresponding 95% confidence intervals within the three IA progression phenotypes pre- and post-SV. **(B)** describes the individual level changes in methylation m-values (y-axis) between the post-SV visit relative to the pre-SV visit in the three IA progression phenotypes (x-axis). Positive values represent increasing DNAm whereas negative values represent decreasing methylation between visits. All DNAm values in **(A, B)** have been adjusted for age, sex, and cell proportions.

Change in methylation at the DNAm site cg16066195 on chr 7 was significantly (FDR adjusted p value=0.0174) different across groups. The reverter group was characterized by an increase in DNAm between pre- and post-SV visits (ie, a positive slope) whereas the progressor and maintainer groups were characterized by no change or a decrease in DNAm (**Figure 2**). This site is an island CpG site (CpG island *chr7:73703458-73704127)* that maps to an area near the *CLIP2* gene.

We also tested whether average DNAm (mean of DNAm levels pre- and post-SV) differed across groups. No DNAm probe was significant at the FDR adjusted alpha level of 0.10.

### 3.3 Differentially methylated region analysis

**Figure 3:**
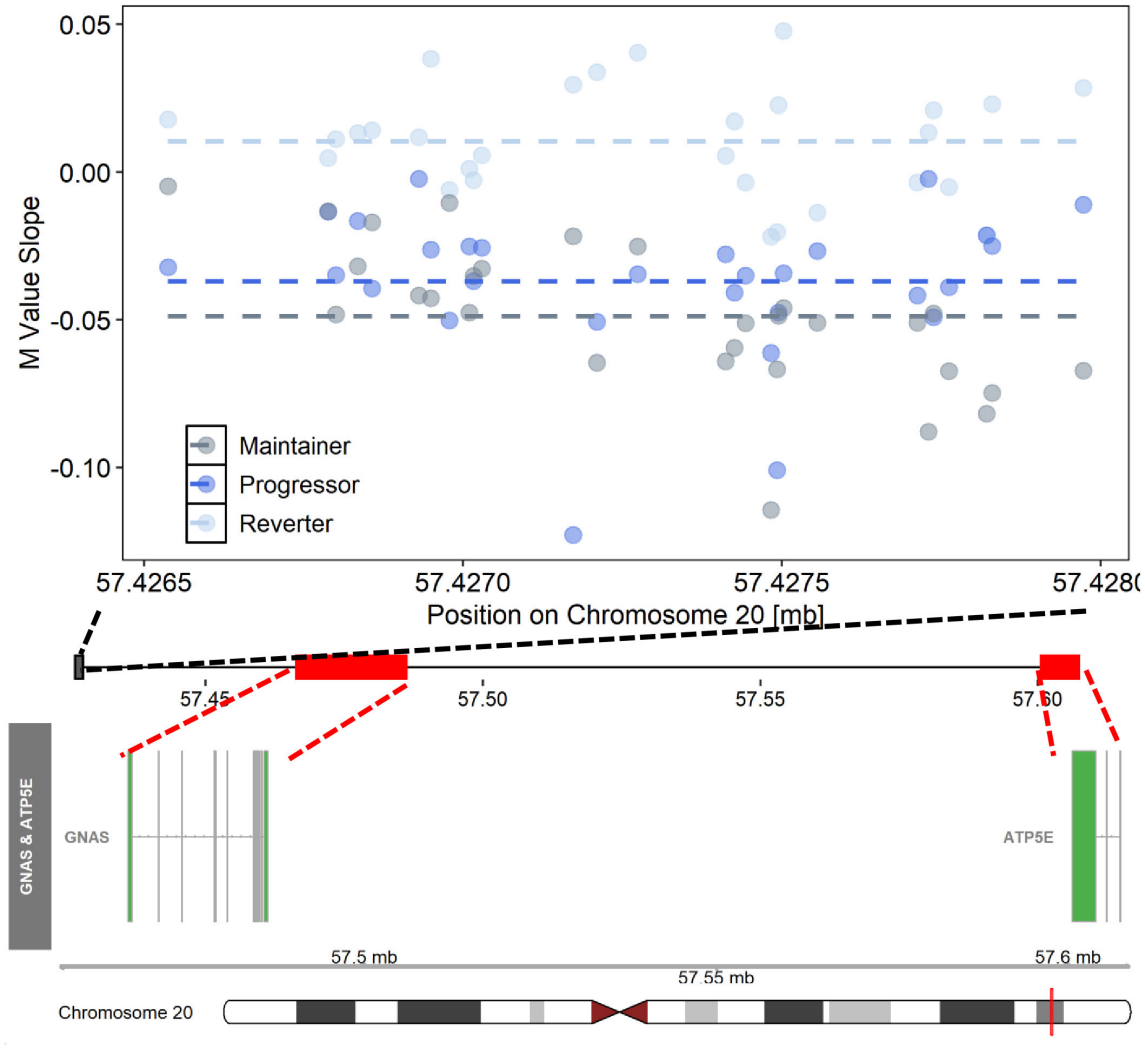
Differentially changing methylation region on chromosome 20 where changes in DNAm (pre- vs post-SV) differed across the three IA progression phenotypes. Description. Region on chromosome 20 loc 57426538 to 57427974 (ΔDMR1) where the change in DNA methylation (DNAm) post- vs pre-SV differed across groups. In the top panel, each dot represents the within group slopes (y-axis) or changes in DNAm m-values between the post-SV and pre-SV visit at each of the CpG sites included ΔDMR 1. The x-axis represents the position (mb) of the CpGs within the region. All slope values were adjusted for age, sex, and cell proportions. Positive values indicate methylation values increased following IA seroconversion whereas negative values indicate methylation decreased following IA seroconversion. The dashed lines represent the average slope value within each group across the entire region. The middle panel represents the location of the region (black solid square) relative to the closest genes, *GNAS* and *ATP5E* (red solid boxes). There are multiple known isoforms for *GNAS* and *ATP5E*, the bottom panel displays the most biologically relevant or consensus transcript based on the Ensembl database. The red line on the ideogram, bottom of the figure, represents the location of *GNAS* and *ATP5E* on chromosome 20.

We also tested for genomic regions (**Figure 1**). In contrast to the single CpG site (DMP) analysis, the regional analysis allowed us to identify multiple CpG sites that demonstrated similar DNAm changes between the pre- and post-SV visits across the three study groups (ΔDMRs). We focused on FDR significant regions of ≥4 DNAm probes where the direction of the change in DNAm (between the pre-SV and post-SV visits) was consistent (100% of probes changed in a similar direction) within one or more of the groups. We identified 11 candidate DMRs (**Table 2**; **Figure 3**).

**Table 2:** Regions where DNAm changes between the post- and pre-SV visits were consistently different across groups (group*visit interaction).

DMR ID	Chr.	Start	Stop	Gene	N CpG Sites	Sidak Adj. Region P	Leading CpG Site	Slope% R*	Slope% P*	Slope% M*	Median Slope R†	Median Slope P†	Median Slope M†
ΔDMR 1	chr20	57426538	57427974	GNAS; GNASAS; GNAS-AS1	29	8.33E-05	cg26496204	69%	100%	100%	0.01	-0.03	-0.05
ΔDMR 2	chr20	36148604	36149751	BLCAP; NNAT	30	1.37E-04	cg24675557	100%	80%	80%	0.05	-0.02	-0.02
ΔDMR 3	chr1	75198582	75199118	TYW3; CRYZ; RP11-17E13.3	8	3.40E-03	cg00121533	100%	88%	100%	0.06	0.04	-0.08
ΔDMR 4	chr14	101291068	101293727	MEG3	25	6.74E-03	cg14034270	96%	85%	100%	0.02	-0.02	-0.02
ΔDMR 5	chr11	1296469	1297386	TOLLIP	7	1.81E-02	cg11095027	86%	57%	100%	0.03	0.03	-0.07
ΔDMR 6	chr15	91473059	91473570	UNC45A	8	2.00E-02	cg03291024	75%	100%	100%	0.01	0.09	-0.09
ΔDMR 7	chr5	1245669	1246292	SLC6A18	4	3.38E-02	cg09075844	100%	100%	100%	-0.03	0.03	-0.06
ΔDMR 8	chr6	170597377	170597899	DLL1	4	3.66E-02	cg05228964	50%	100%	100%	<0.01	0.10	-0.04
ΔDMR 9	chr6	28945322	28945493	RN7SL471P‡	4	6.09E-02	cg10919664	100%	100%	100%	0.07	0.06	-0.16
ΔDMR 10	chr6	27647713	27648355	RP1-15D7.1‡	4	7.14E-02	cg25106913	75%	75%	100%	<0.01	0.06	-0.05
ΔDMR 11	chr5	1867978	1868694	IRX4‡	6	8.71E-02	cg14773178	83%	100%	100%	0.04	0.08	-0.08

DMRs limited to regions with a minimum of 4 probes and 100% of within group slopes in the same direction for one or more groups.

Chr., chromosome.

Start/Stop, DMR start and stop position.

Gene, Gene annotation from the Illumina manifest file, based on UCSC reference genes mapped to CpG sites within DMR and/or genes mapped to CpG sites within known regulatory regions, if gene was not annotated within the Illumina manifest file, noted with ^‡^, gene name based on closest transcription start site.

Leading CpG site, most significant DMP within the region.

Sidak Adj. Region P, regional p value corrected for multiple testing based on number similarly sized regions possible based on genomic coverage in the DMR analysis.

*R, reverters; P, progressors; M, maintainers, Percent of within group slopes (Pre-SV vs Post-SV) in the same direction (hypo (–) or hyper (+) methylation) across all the probes included in the DMR.

**^†^** Median slope (Pre-SV vs Post-SV) across all probes included in the DMR for each group, (+) values indicate increasing DNAm (–), indicate decreasing DNAm.

**Figure 4:**
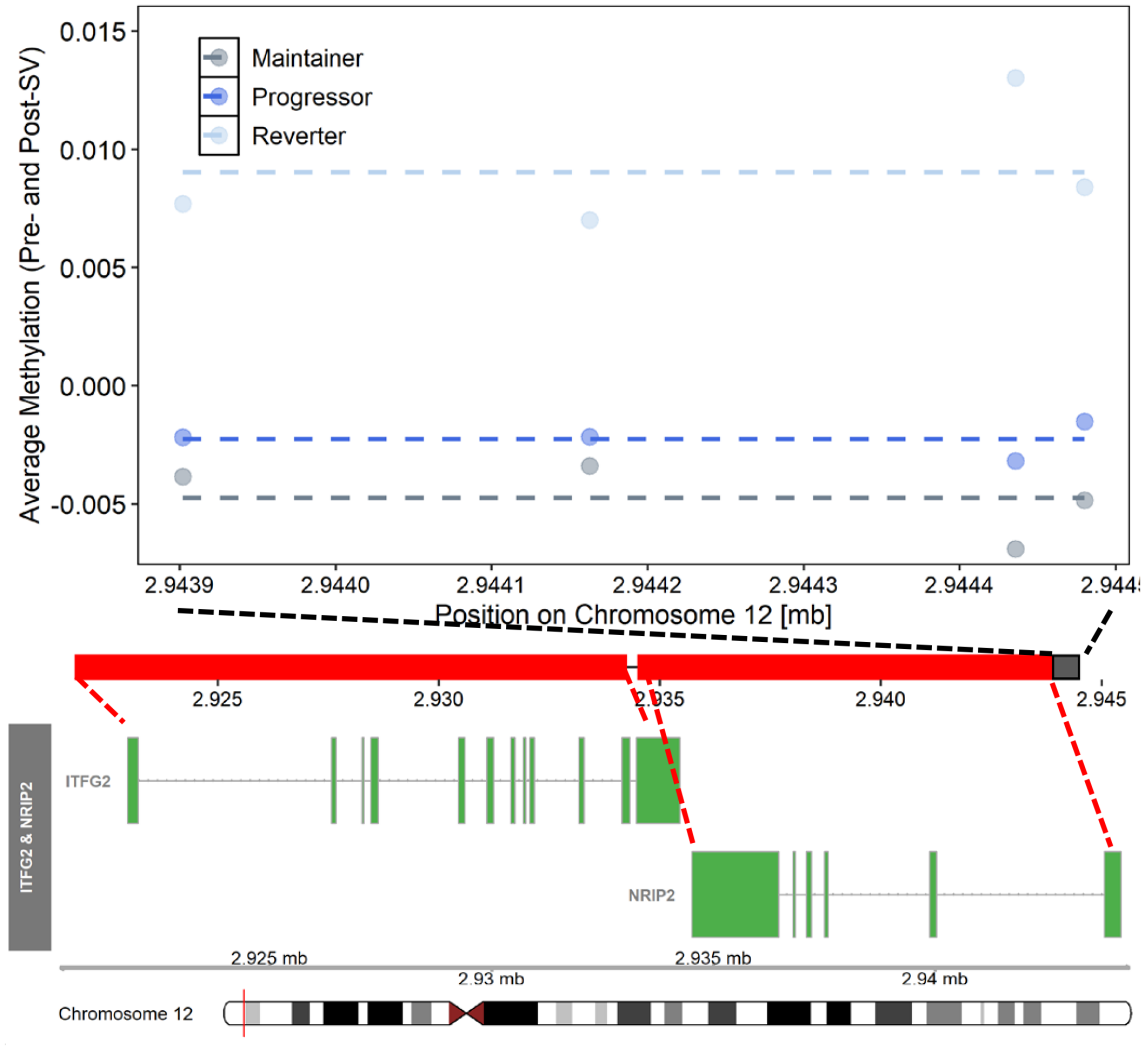
Differentially methylated region on chromosome 12 where average (pre- and post-SV) methylation levels differed across the three IA progression phenotypes. Description. Region on chromosome 12 loc 2943902 to 2944481 (μDMR4) where average DNA methylation (DNAm) levels, post- and pre-SV, differed across groups. In the top panel, each dot represents the average DNAm value (y-axis) at each of the CpG sites included μDMR4. The x-axis represents the position (mb) of the CpGs within the region. All DNAm values were adjusted for age, sex, cell proportions, and genetic ancestry. The dashed lines represent the average methylation value within each group across the entire region. The middle panel represents the location of the region (black solid square) relative to the closest genes, *ITFG2* and *NRIP2* (red solid squares). There are multiple known isoforms for *ITFG2* and *NRIP2*, the figure displays the most biologically relevant or consensus transcript based on the Ensembl database. The red line on the ideogram, bottom of the figure, represents the location of *ITFG2* and *NRIP2* on chromosome 12.

We also tested for regions where the average DNAm levels at the pre- and post-SV visits differed across the groups (μDMRs). We identified 22 FDR significant μDMRs of ≥4 DNAm probes where the direction of the pairwise group differences in DNAm was consistent across all CpG sites included in the region (**Table 3**; **Figure 4**).

**Table 3:** Regions where average of post- and pre-SV DNAm levels were consistently different across groups (group main effect).

DMR ID	Chr.	Start	Stop	Gene	N Probes	Sidak Adj. Region P	Leading CpG Site	Median PvR‡	Median RvM‡	Median PvM‡
μDMR 1	chr1	180922636	180923341	RP11-46A10.4; RP11-46A10.5	4	1.38E-05	cg00579423	0.09	0.37	0.46
μDMR 2	chr10	99338056	99338241	ANKRD2	4	1.75E-04	cg27469738	-0.11	0.26	0.17
μDMR 3	chr10	52008360	52008906	ASAH2	4	6.45E-03	cg24123634	-0.07	-0.02	-0.11
μDMR 4	chr12	2943902	2944481	NRIP2; ITFG2	4	7.06E-03	cg02852959	-0.15	0.19	0.04
μDMR 5	chr12	75784855	75785098	GLIPR1L2; CAPS2	6	7.59E-03	cg12351126	0.10	0.24	0.34
μDMR 6	chr12	51566379	51567113	TFCP2	7	1.24E-02	cg19016289	0.05	0.15	0.2
μDMR 7	chr1	1289835	1290713	MXRA8	6	1.61E-02	cg07284273	-0.16	0.33	0.15
μDMR 8	chr15	72766637	72767333	ARIH1; RP11-1007O24.3	4	1.93E-02	cg26880891	0.09	0.02	0.14
μDMR 9	chr19	45206843	45207560	CEACAM16	4	2.78E-02	cg24091949	-0.09	-0.04	-0.13
μDMR 10	chr19	2250901	2251068	AMH	4	2.83E-02	cg23218559	-0.18	0.38	0.21
μDMR 11	chr18	7567426	7568266	PTPRM	5	3.44E-02	cg05870479	0.09	0.04	0.11
μDMR 12	chr15	85524778	85525674	PDE8A	4	4.02E-02	cg02839273	0.05	0.05	0.13
μDMR 13	chr2	85765644	85766105	MAT2A	4	4.39E-02	cg06978067	0.08	0.05	0.13
μDMR 14	chr19	48048129	48049234	ZNF541	4	4.90E-02	cg22341310	-0.12	0.17	0.06
μDMR 15	chr4	4861683	4862241	MSX1	4	5.94E-02	cg11930592	0.12	-0.04	0.08
μDMR 16	chr11	598325	599091	PHRF1	5	7.14E-02	cg12921473	-0.06	-0.05	-0.10
μDMR 17	chr5	101119084	101119767	OR7H2P*	4	7.67E-02	cg12197752	0.09	0.18	0.29
μDMR 18	chr13	42031761	42032737	C13orf15; RGCC	4	8.16E-02	cg18495682	0.06	0.02	0.09
μDMR 19	chr3	38206610	38207525	OXSR1	4	8.20E-02	cg19728055	0.07	0.05	0.11
μDMR 20	chr10	14372431	14372914	FRMD4A	5	8.45E-02	cg05755354	-0.16	-0.02	-0.18
μDMR 21	chr8	145550361	145551157	DGAT1	5	8.72E-02	cg11127482	0.06	0.04	0.11
μDMR 22	chr11	128693473	128694916	FLI1*; KCNJ1*	9	9.44E-02	cg15509024	-0.12	-0.09	-0.18

DMRs limited to regions with a minimum of 4 probes and direction of pairwise comparison was consistent across all probes in the region.

Chr., chromosome.

Start/Stop, DMR start and stop position.

Sidak Adj. Region P, regional p value corrected for multiple testing based on number similarly sized regions possible based on genomic coverage in the DMR analysis

Leading CpG site = most significant DMP within the region

Gene, Gene annotation from the Illumina manifest file, based on UCSC reference genes mapped to CpG sites within DMR and/or genes mapped to CpG sites within known regulatory regions, if gene was not annotated within the Illumina manifest file, noted with *gene name based on closest transcription start site.

^‡^R, reverters; P, progressors; M, maintainers, Median effect size across the region representing difference in methylation M values between groups.

### 3.4 eQTM candidate prioritization

We tested the correlation between DNAm and cis- gene expression levels in a subset of overlapping samples. The availability of individual level DNAm data allowed us to look at the entire DMR together. Based on the ΔDMR candidates, we identified two FDR significant cis eQTMs representing one DMR and two gene transcripts, GNAS and ATP5E (ΔDMR1, region on chromosome 20, see **Table 4**). Within this region, increased DNAm post- vs pre-SV was positively associated with expression of GNAS and ATP5E (see **Table 4**).

**Table 4:** Summary of FDR significant cis-eQTMs representing correlation between differentially changing methylation regions and gene expression post- SV.

Methylation DMR Information					Cis-Gene Expression Information						
DMR ID	Chr.	DMR Start	DMR Stop	N Probes	Gene Symbol	Ensembl ID	Strand	Gene Start	Gene End	Corr*	FDR
ΔDMR 1	20	57426538	57427974	29	GNAS	ENSG00000087460	1	57414773	57486247	0.559	0.0667
ΔDMR 1	20	57426538	57427974	29	ATP5E	ENSG00000124172	-1	57600522	57607437	0.557	0.0667

DNAm levels for all probes identified in the DMR analysis (**Tables 1**, **2**) were included in a PCA. We then tested the association between the 1st PC and RNA seq data from overlapping visit at the post-SV visit. Only significant cis (TSS +/- 500KB of midpoint of DMR) expression quantitative trait methylation (cis-eQTM) associations are presented.

*Spearman correlation coefficient.

Chr., chromosome.

DMR Start/End, DMR start and end position.

Gene Start/End, Gene start and end positions (based on annotation file for GEO, GSE50244).

Beta, beta coefficient from linear regression model (adjusted for age and sex) representing association between 1st PC from DNAm probes in each DMR and islet cell pancreas expression.

FDR, Benjamini-Hochberg FDR adjusted p value.

### 3.5 Metabolite quantitative trait methylation analysis candidate prioritization in overlapping samples

**Figure 5:**
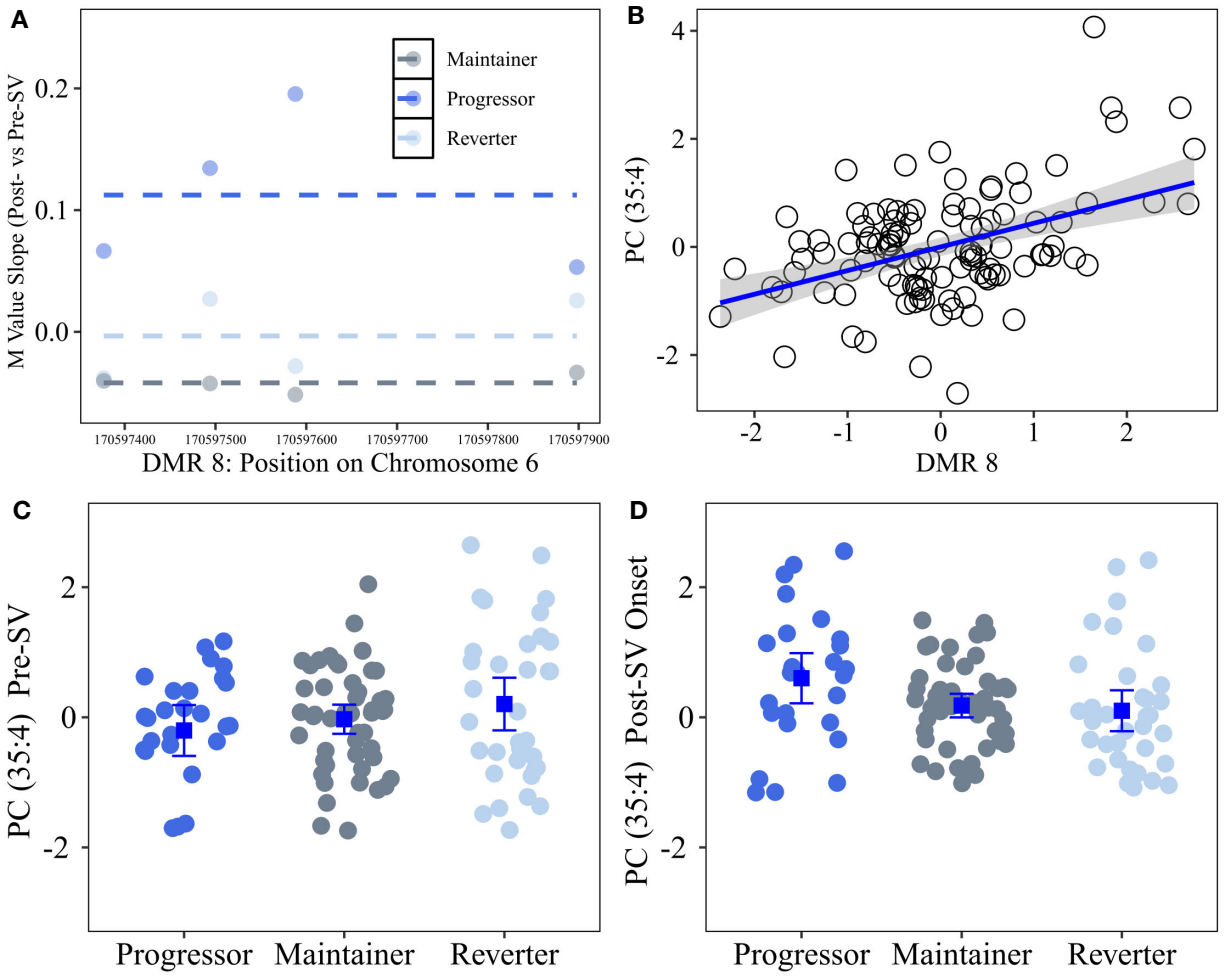
Differentially changing region on chromosome 6 (post- vs pre-SV) that was positively correlated with changes in lipid metabolites (post- vs pre-SV). Description: Region on chromosome 6 loc 170597377 to 170597899 (ΔDMR8) where the change in DNA methylation (DNAm) post- vs pre-SV differed across groups. In the top left **(A)**, each dot represents the within group slopes (y-axis) or changes in methylation m-values between the post-SV and pre-SV visit at each of the CpG sites included ΔDMR 8. The x-axis represents the position (mb) of the CpGs within the region. Positive values indicate DNAm values increased following IA seroconversion whereas negative values indicate DNAm decreased following IA seroconversion. The dashed lines represent the average slope value within each group across the entire region. The top right **(B)** represents the association between DMR wide DNAm captured by the 1^st^ PC (x-axis) and changes in metabolite values (y-axis) between the post- and pre-SV visits. DNAm and metabolite expression values have been standardized to facilitate the interpretation of the slope as a 1 standard deviation increase in the change in metabolite levels between the post- and pre-SV visits per 1 standard deviation increase in the change in methylation between post- and pre-SV visits. The bottom panels **(C, D)** represent the average metabolite levels and corresponding 95% confidence intervals within the three groups pre- and post-SV. All DNAm and metabolite values were adjusted for age, sex, and cell proportions.

We tested whether the single DMP candidate, cg16066195, as well as the candidate DNAm regions identified in our primary analysis were associated with metabolite levels. Consistent with the eQTM analysis, we regressed out age and sex from annotated metabolites and then tested the correlation between annotated metabolites versus DNAm regional PCs. Based on the ΔDMR candidates, we identified 26 annotated metabolites from the Lipid panel that were correlated with 4 DMRs (see **Table 5**; **Figure 5**). ΔDMR 8 was correlated with multiple lipids, primarily PCs, ΔDMR 5 was also correlated with multiple lipids, primarily correlated with TGs (fats). ΔDMR 9 and ΔDMR 2 were correlated with a single lipid, an ether lipid, and a TG, respectively. Metabolite candidates primarily consisted of odd-chain fatty acid containing lipid species (OCFA). Furthermore, the majority of the metabolites (29/30) were positively correlated with increasing DNAm levels. The μDMR candidate regions as well as the single DMP candidate were not significantly associated with metabolite levels at our FDR adjusted cutoff of 0.10.

**Table 5:** Secondary metQTM analysis of the association between pre- versus post-SV change in methylation across the DMRs and pre- versus post-SV change in metabolite levels.

DMR ID	Chr.	DMR Start	DMR Stop	Metabolite Name†	Standardized Beta	FDR Adj. P Value
ΔDMR 2	chr20	36148604	36149751	TG (49:2)	0.320	0.0992
ΔDMR 5	chr11	1296469	1297386	TG (53:2)	0.411	0.0121
				Phosphatidylcholine (33:1)	0.361	0.0469
				TG (53:3)	0.353	0.0627
				PE (38:4)	0.339	0.0826
				TG (49:2)	0.330	0.0948
				TG (47:0)	0.329	0.0952
				TG (51:3)	0.327	0.0954
				PC (33:1)	0.327	0.0954
				Phosphatidylcholines (35:1)	0.325	0.0954
				TG (53:1)	0.320	0.0992
ΔDMR 8	chr6	170597377	170597899	Phosphatidylcholine (35:4)	0.438	0.0078
				Phosphatidylcholines (33:1)	0.404	0.0121
				Phosphatidylcholines (33:0)	0.403	0.0121
				Phosphatidylcholines (33:1)	0.402	0.0121
				Phosphatidylcholines (35:3)	0.396	0.0138
				LPC (15:0)	0.393	0.0139
				Phosphatidylcholines (38:5)	0.375	0.0527
				Phosphatidylcholines (33:2)	0.366	0.0445
				Phosphatidylcholines (35:4)	0.365	0.0445
				Phosphatidylcholines (31:0)	0.350	0.0647
				Phosphatidylcholines (35:1)	0.347	0.0647
				Phosphatidylcholines (36:3)	0.347	0.0647
				Phosphatidylcholines (p-34:0) or Phosphatidylcholines (o-34:1)	-0.334	0.0940
				TG (49:3)	0.332	0.0940
				Phosphatidylcholines (33:2)	0.332	0.0940
				Phosphatidylcholines (36:3) B	0.325	0.0954
				Phosphatidylcholines (37:6)	0.324	0.0954
				Phosphatidylcholines (35:1)	0.323	0.0975
ΔDMR 9	chr6	28945322	28945493	*Phosphatidylcholine* (p-38:2) or *Phosphatidylcholine*(o-38:3)	0.345	0.0662

DNAm levels for all probes identified in the DMR analysis (**Tables 1**, **2**) were included in a PCA. We then tested the association between the 1st PC changes in metabolites between the pre- and post-SV visits.

Chr., chromosome.

DMR Start/End, DMR start and end position.

Gene Start/End, Gene start and end positions (based on annotation file for GEO, GSE50244).

Standardized Beta, beta coefficient from linear regression model testing the association between change in DNAm and change in metabolites pre-SV vs post-SV. The slopes have been standardized to represent a 1 stdev change in metabolite per 1 standard deviation change in DNAm regional PC levels.

FDR Adj. P value, Benjamini-Hochberg FDR adjusted p value.

^†^See **Appendix 5 (Data Sheet 5) (Tables D, E)** for complete annotation for all metabolites included in **Table 5**.

## 4 Discussion

Epigenetic biomarkers are appealing in the study of complex diseases such as T1D based on their heritability, role in gene expression, and responsiveness to external stimuli. Epigenetic effects in observational studies are challenging to interpret because it is often not possible to determine whether DNA methylation (DNAm) is causative or secondary to the disease process. A strength of our study is the longitudinal analysis of DNAm levels both before and after the onset of IA. We identified a single CpG site as well as genomic regions where changes in DNAm between the post-SV and pre-SV visits were significantly different across the IA progression phenotypes. We also identified regions where average DNAm levels pre- and post-SV differed across the progression phenotypes. Together, the DNAm regions have potential biological relevance to T1D etiology based on their potential role in immune and beta cell function.

We identified a DNAm site, cg16066195, on chromosome 7 where DNAm levels increased between the pre- and post-SV visits among individuals who reverted to an IA negative state (reverters) compared to progressors (who showed no change in DNAm) and maintainers (who showed decreasing DNAm, **Figure 2**). This island CpG is located near the transcription start site for the protein coding gene *CLIP2*. In a mouse model of diet induced changes in beta cell expression, *CLIP2* gene expression was significantly downregulated among mice fed a carbohydrate containing diabetogenic high-fat diet relative to mice fed a diabetes-protective carbohydrate free high-fat diet (39). Furthermore, SNPs within *CLIP2* (rs2528994 and rs512023) have demonstrated modest associations with T2D in both the Diabetes Genetics Initiative (40) and the Wellcome Trust Case Control Consortium (41).

Our methylation analysis also identified numerous regions where average methylation post- and pre-SV differed across the autoimmune phenotypes in areas of the genome potentially relevant to T1D etiology. We identified a DMR on chromosome 12, μDMR4, characterized by hypermethylation in the reverter group relative to the progressor and maintainer groups (**Figure 4**). This includes 4 probes that, based on the ENCODE Project Consortium (42), are located in a known enhancer region. Three of the four probes within this region are located within the transcription start site for *NRIP2*, predicted to act upstream or within the notch signaling pathway (43). This pathway is relevant to T1D (44) based on its role in immune cell differentiation and function (45) as well as pancreas development (46), islet cell function (47), and islet cell survival (48). All four probes within μDMR4 are also located within the 5’UTR region for *ITFG2*, a gene expressed in numerous tissues including immune cells. Mouse and *in vitro* models have demonstrated that *ITFG2* deficiency alters B cell maturation and migration (49). In a lupus mouse model, MRL/lpr, autoimmunity development occurred earlier and was more severe in *ITFG2* deficient mice (49). Together, these findings suggest a potential role for *ITFG2* in B cell differentiation and as a potential regulator of autoimmunity. Although, average methylation within DMR4 was not correlated with expression of ITFG2 or NRIP2 in our secondary eQTM analysis, three probes within μDMR4 (cg05194726; cg06997549; cg02852959) were correlated with expression of both ITFG2 and NRIP2 in whole blood based on the BIOS QTL browser (50), an online resource that provides a searchable database of FDR significant associations between DNAm and gene expression (eQTM). Additional work is needed to understand the connections between methylation within this region on chr 12, ITFG2 expression, NRIP2 expression, and T1D etiology.

We also identified several regions of differentially changing DNAm that are potentially relevant to T1D etiology based on known associations between DNAm in these regions and relevant environmental risk factors. We identified a region on chr 20 near the *GNAS/GNASAS* loci, ΔDMR 1, that was characterized by decreasing DNAm pre- vs post-SV in maintainers and progressors relative to reverters (**Table 2**; **Figure 3**). Based on the ENCODE Project Consortium (42), 25 of the 29 probes in ΔDMR 1 are located within a DNAase hypersensitivity region and 4 probes are known to interact with transcription factor binding. DNAm in this region is responsive to environmental stressors. Umbilical cord blood DNAm near *GNAS* was altered among infants born to a mother affected by gestational diabetes (GDM), a disorder characterized by glucose intolerance during pregnancy (51). Based on the Dutch Hunger Winter Families Study (52), siblings exposed to the war-time Dutch Hunger Winter famine were associated with persistent changes in DNAm in a region near the *GNASAS* locus relative to their unexposed siblings (53). The direction and magnitude of effect depended on timing of exposure and sex of the exposed individual (53). DNAm among exposed siblings was also altered near another gene implicated in metabolic disease *MEG3* (53), a gene that mapped to ΔDMR4 which was also characterized by decreasing methylation among progressors and maintainers relative to reverters (**Table 2**). Interestingly, both the GNAS (54) and MEG3 (55) genes are maternally imprinted. Loss of maternal imprinting should be investigated as a potential mechanism in the etiology of T1D using whole-genome bisulfite sequencing in order to provide a higher density representation of DNAm changes within imprinted areas of the genome.

The secondary eQTM analysis in a subset of overlapping samples confirmed that changes in methylation within ΔDMR1 were associated with expression of GNAS. Increased methylation post- versus pre-SV was associated with higher levels of GNAS expression at the post-SV visit in a subset of overlapping samples. GNAS is an important regulator of insulin secretion in beta cells (56). GNAS silencing results in decreased insulin secretion and insulin content (56). GNAS encodes the G protein subunit alpha which also plays a role in the interaction between antigen presenting cells and T helper cell differentiation (57). Mice with dendritic cells deficient for GNAS result in a phenotype characterized by preferential Th2 differentiation, Th2 type inflammation, and subsequent development of allergic asthma (57). Overlap between autoimmunity and atopic conditions have long been hypothesized based on disruptions in similar immune pathways (58). Positive associations between childhood asthma and subsequent T1D development have been observed in several countries (59–61). Overall, our results suggest that maintenance of DNAm levels near GNAS during IA may represent a unique protective mechanism in reverters.

In order to further characterize the DNAm regions identified in the primary analysis, we tested the correlation between changes in DNAm and changes in annotated metabolites (metQTM). Four differentially changing DMRs were correlated with changes in 26 unique lipid metabolites (**Table 5**). ΔDMR 8, characterized by increasing methylation in progressors (**Figure 5**), was correlated with 18 of the 26 lipid metabolites. This region of differentially changing methylation is notable based on its location in an open chromatin region within the body of the *DLL1* gene on chr. 6. As a notch signaling ligand, DLL1 controls the differentiation of pancreatic progenitor cells into exocrine versus endocrine cells (46). The loss of DLL1 results in early progenitor cell differentiation and an overabundance of endocrine cells (46). A recent mouse model confirmed DLL1 is also relevant to islet cell function in the mature pancreas based on its high level of expression in beta cells and corresponding role in insulin secretion (47). Furthermore, DLL1 plays an important role in differentiation of B cells and the development of antigen secreting cells; the presence of DLL1 influences AB titer levels and isotype switching (45). Additional work is needed to understand the connection between a concordant increase in lipid levels and DNAm within the *DLL1* gene following seroconversion.

Our secondary metQTM was unique in that DNAm and metabolite levels were available pre- and post-SV in a subset of overlapping samples. This analysis revealed a consistent positive association between increasing lipid metabolite levels, post- vs pre-SV, and increasing DNAm levels across several regions (25 of the 26 unique lipid metabolites were positively correlated with DNAm changes, see **Table 5**). Numerous studies (62–68) have reported associations between dysregulation in lipid levels and T1D. Although lipid levels have been shown to be influenced by age at sample collection/timing of sample collection relative to onset of IA and type of first appearing autoantibody, prior research suggests lower lipid levels, including sphingomyelins and phosphatidylcholines, are generally associated with increased risk of T1D and/or IA (62–68). In our study, increasing lipid levels, in particular phosphocholines, following the onset of IA were strongly correlated with increasing methylation within ΔDMR8. This region was characterized by increasing methylation within the progressor group. However, as demonstrated in **Figure 5**, the lipid metabolite most strongly correlated with DNAm changes in this region, *Phosphatidylcholine* (35:4), was lower in the progressor group prior to SV and then subsequently increased following the onset of IA, suggesting higher levels of lipids within the progressor group may be unique to changes that occur following seroconversion.

There was a high prevalence of odd-chain fatty acid (OCFA) containing lipid species among the metabolites correlated with DNAm changes. Recently, there has been increased recognition of OCFA in plasma and their potential biological relevance (69). OCFA levels have been associated with glucose homeostasis, insulin resistance, T2D, and BMI (69, 70). Pfleuger et al (71) observed higher levels of odd-chain triglycerides among autoantibody positive versus negative children in BABYDIAB. This parallels the concordant post-seroconversion increase in OCFA levels and DNAm near the DLL1 gene (ΔDMR 8) among progressors (**Figure 5**) in the current study. OCFA have been proposed a marker of dairy intake which has been positively correlated with progression to T1D in prior work in DAISY (72). However, dairy intake contributes modestly to OCFA levels. These lipids primarily originate endogenously from adipocytes as well as from dietary intake of numerous foods including dairy, poultry, and fiber (70, 73, 74). Additional work is needed to understand connections between increasing methylation and increasing OCFA as well as the source of these lipid species.

A major strength of our study was the inclusion of DNAm measurements prior to T1D as well as the multi-omics work used to identify correlations between DNAm and gene expression as well metabolite levels. We measured DNAm before and after SV (ie, the appearance of IA) which builds on prior studies that have included DNAm measures after T1D and/or after IA onset only (14–16). A novel feature of our longitudinal methodology was our group*visit interaction modelling strategy that allowed us to identify changes in DNAm before and after the onset of IA, a critical window in T1D pathogenesis. These within individual effects are essential to understanding the etiology of T1D as they are robust to individual level confounders such as sex, genetic predisposition, and/or family history. Johnson et al (17) also used a longitudinal case-control analysis of T1D cases vs. unaffected controls in DAISY. In contrast, the current study design focused on individuals who developed IA and furthermore, tested for differences in DNAm post- vs pre-SV (group*visit interaction) rather than testing for differences in methylation by age (group*age interaction). Comparing the DMRs identified by this study versus Johnson et al (17), only two regions were located within 1 MB of each other–one on chr 6 ΔDMR 9 (28945322–28945493) in the current study vs chr 6 28973328-28973521 in Johnson et al (17), and one on chr 20 ΔDMR 2 (36148604–36149751) in the current study vs chr 20 36148954-36149232 in Johnson et al (17). Consistent with prior work, ΔDMR 9 and ΔDMR 2 were both associated with differential changes in DNAm in progressors relative to maintainers and/or reverters.

### 4.1 Limitations

We obtained DNAm from whole blood, which means we were unable to identify cell subtype specific effects. Similarly, our study focused on blood tissue only. DNAm changes within the blood may not reflect DNAm changes within other tissues that contribute to T1D, such as the pancreas. Due to advancements in technology during the study, DNAm was measured on two platforms. Individuals were randomly assigned to the platforms to minimize bias. We looked for cis-eQTMs. Given that it is possible that regions act over larger areas of the genome, we may have missed larger effects that occurred outside of our 500 KB window. Due to the small sample size, the eQTM was underpowered to identify FDR significant DMR vs gene transcript pairs. This limitation may explain lack of concordance between eQTM results and BIOS QTL results (μDMR4). Furthermore, among the two gene transcripts that were correlated with changes in methylation within ΔDMR1, gene expression data were only available at the post-SV visit. Therefore, it was not possible to determine whether gene expression also changed pre- versus post-SV. Finally, metabolite levels are influenced by age and dietary patterns. Although we adjusted for age, the large differences in age between the progressor group and the reverter and maintainer groups creates challenges in interpreting the metabolite vs methylation correlations. Additional work is need to replicate the metabolite vs DNAm regional effects.

## 5 Conclusion

T1D is an autoimmune disease characterized by immune mediated destruction of beta cells. Beta cell stress has been proposed as a mechanism connecting environmental perturbations such as infection, inflammation, diet, and increased insulin secretion to disease progression (75). Our EWAS identified DNAm candidates known to be modified by diabetes relevant environmental factors including diet and glucose levels (*CLIP2*, *GNAS/GNAS-AS, MEG3*). Our results also implicated genes (*DLL1* and *GNAS*) with functional roles in both beta and immune cells. Our results build upon prior work by identifying specific areas of the genome where DNAm changes pre- and post-SV visits differentiated between reversion versus progression of IA. The correlation between changes in DNAm and changes in lipid levels reveal common connections between DMRs in different areas of the genome that may be related to disruptions in lipid metabolic pathways. Additional work is needed to replicate these findings, test for cell-specific changes in DNAm pre- vs post-seroconversion, and to identify modifiable factors that lead to these DNAm changes; ideally, the first step in the development of preventative strategies that delay or prevent progression of IA.

## Data availability statement

The datasets presented in this study can be found in online repositories. The names of the repository/repositories and accession number(s) can be found below: https://www.ncbi.nlm.nih.gov/, PRJNA597238.

## Ethics statement

The studies involving humans were approved by Colorado Multi-institutional Review Board. The studies were conducted in accordance with the local legislation and institutional requirements. Written informed consent for participation in this study was provided by the participants’ legal guardians/next of kin.

## Author contributions

PC: Writing – review & editing, Writing – original draft, Project administration, Methodology, Formal analysis, Conceptualization. LV: Writing – review & editing, Software, Methodology, Data curation. RJ: Writing – review & editing, Methodology, Data curation. TB: Writing – review & editing. AS: Writing – review & editing, Supervision. KK: Writing – review & editing, Supervision, Methodology. IY: Writing – review & editing, Supervision. TF: Writing – review & editing, Supervision. OF: Writing – review & editing, Resources, Data curation. MR: Writing – review & editing, Supervision, Resources, Funding acquisition. JN: Writing – review & editing, Supervision, Methodology, Funding acquisition, Conceptualization.
