# Supplementary material for: Longitudinal changes in DNA methylation during the onset of islet autoimmunity differentiate between reversion versus progression of islet autoimmunity
Source: Front Immunol. 2024 Jun 10;15:1345494. doi: 10.3389/fimmu.2024.1345494 (PMC11194352; doi:10.3389/fimmu.2024.1345494)
Supplement: Supplementary file 1 [file DataSheet_1.pdf]

#Interaction Models

```
Long.MM.Fit <- lme (M ~  Group + Visit + Group*Visit + Female_YN + Age + CD8T +           CD4T +  NK +
            Bcell +   Mono + Dataset, random=~1|ID,
                    control = lmeControl(maxIter = 1e8, msMaxIter = 1e8), method = "REML",
                    na.action=na.omit)
```

#Group effect models

```
Long.MM.Fit <- lme (M ~  Group + Female_YN + Age + CD8T +     CD4T +  NK +      Bcell +   Mono + Dataset
+ PC1 + PC2 + PC2, random=~1|ID,
                    control = lmeControl(maxIter = 1e8, msMaxIter = 1e8), method = "REML",
                    na.action=na.omit)
```

#Group = IA group (Reverter, Progressor, Maintainer)

#Female_YN = (1 = Female, 0 Male)

#Age = (age at visit | 2 visits per subject)

#CD8T = estimated CD8T cell proportion

#CD4T = estimated CD4T cell proportion

#Bcell = estimated B Cell cell proportion

#Mono = estimated monocyte cell proportion

#NOTE: granulocytes are used as the reference group, and thus are left out of the model

#PC1 = genetic ancestry PC1

#PC2 = genetic ancestry PC2

#ID = subject level identifier

#Dataset = methylation platform (450K vs 850K)
