# Supplementary material for: Longitudinal changes in DNA methylation during the onset of islet autoimmunity differentiate between reversion versus progression of islet autoimmunity
Source: Front Immunol. 2024 Jun 10;15:1345494. doi: 10.3389/fimmu.2024.1345494 (PMC11194352; doi:10.3389/fimmu.2024.1345494)
Supplement: Supplementary file 2 [file DataSheet_2.docx]

**Appendix 2.** Test statistic inflation


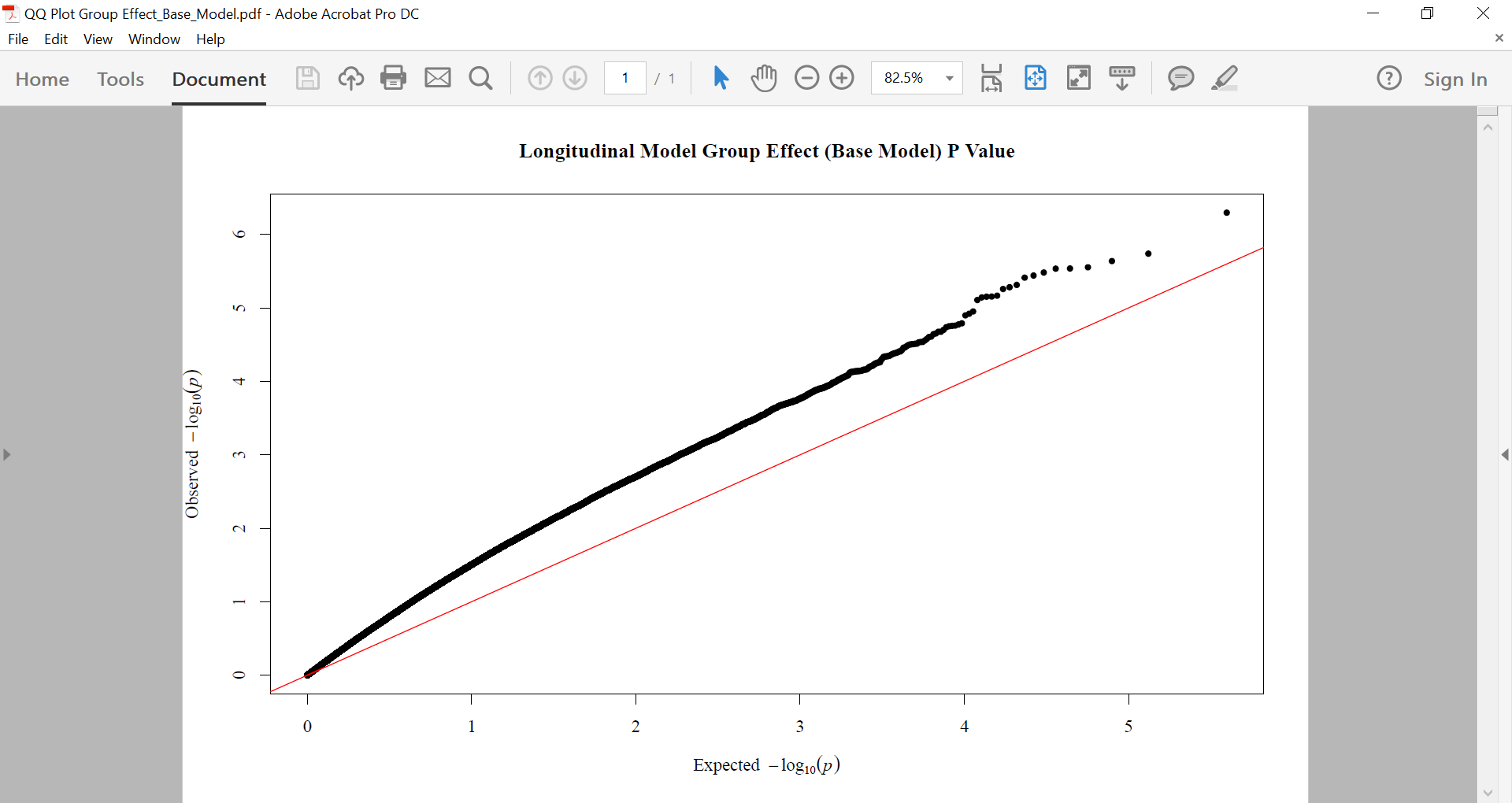

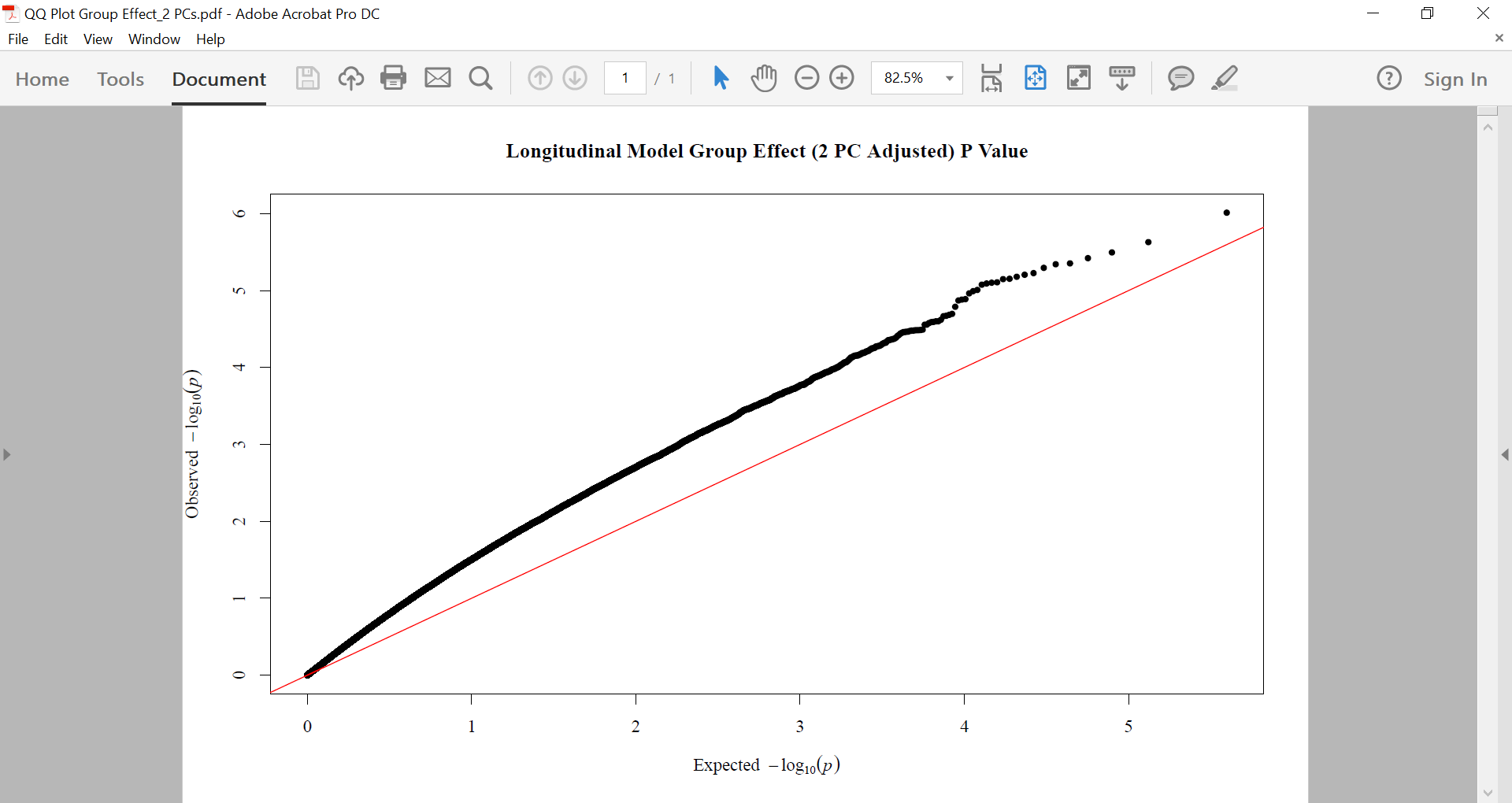


**Appendix Figure A (Left) and B (Right):** Genomic inflation and model Diagnostics for the group main effect longitudinal model. We used two models, a group main effect model (**Appendix Figure A and B**) and a group*visit interaction model (**Appendix Figure B**), to test the association between methylation levels and the three islet autoimmunity progression phenotypes. The group variable was a three-level variable consisting of reverters (developed IA, reverted to an IA negative state and was negative for all autoantibodies at the final visit), progressors (developed IA and progressed to T1D), maintainers (developed IA and was positive for one or more autoantibodies at the final study visit). Visit was a two-level variable representing the two study visits, pre-IA and post-IA. The group effect model tested the null hypothesis that the average of the pre-IA and post-IA methylation levels did not differ across the IA progression phenotypes. Review of the Q-Q plot from the base model (**Appendix Figure A**) identified possible presence of genomic inflation. Therefore, we considered addition of ancestral PC data based on exome chip data obtained under separate funding (see methods section, Population Ancestry). We considered including up to 5 ancestral PCs. The model including 2 PCs (**Appendix Figure B**) was associated with lowest lambda values based on traditional lambda calculation as well as the lambda value calculated using the Bacon R package bacon (v1.16.0) R package^1^. Traditional approaches tend to overestimate lambda values in epigenetic association studies^1^. The bacon method is purported to provide a better measure of inflation in these studies^1^.

| **Appendix Table A.** Genomic inflation lambda values | | |
| --- | --- | --- |
| Number of PCs | Lambda | Lambda (Bacon) |
| 0 | 2.22 | 1.00 |
| 1 | 2.22 | 1.00 |
| 2 | 2.17 | 1.00 |
| 3 | 2.18 | 1.01 |
| 4 | 2.17 | 1.32 |
| 5 | 2.17 | 1.32 |


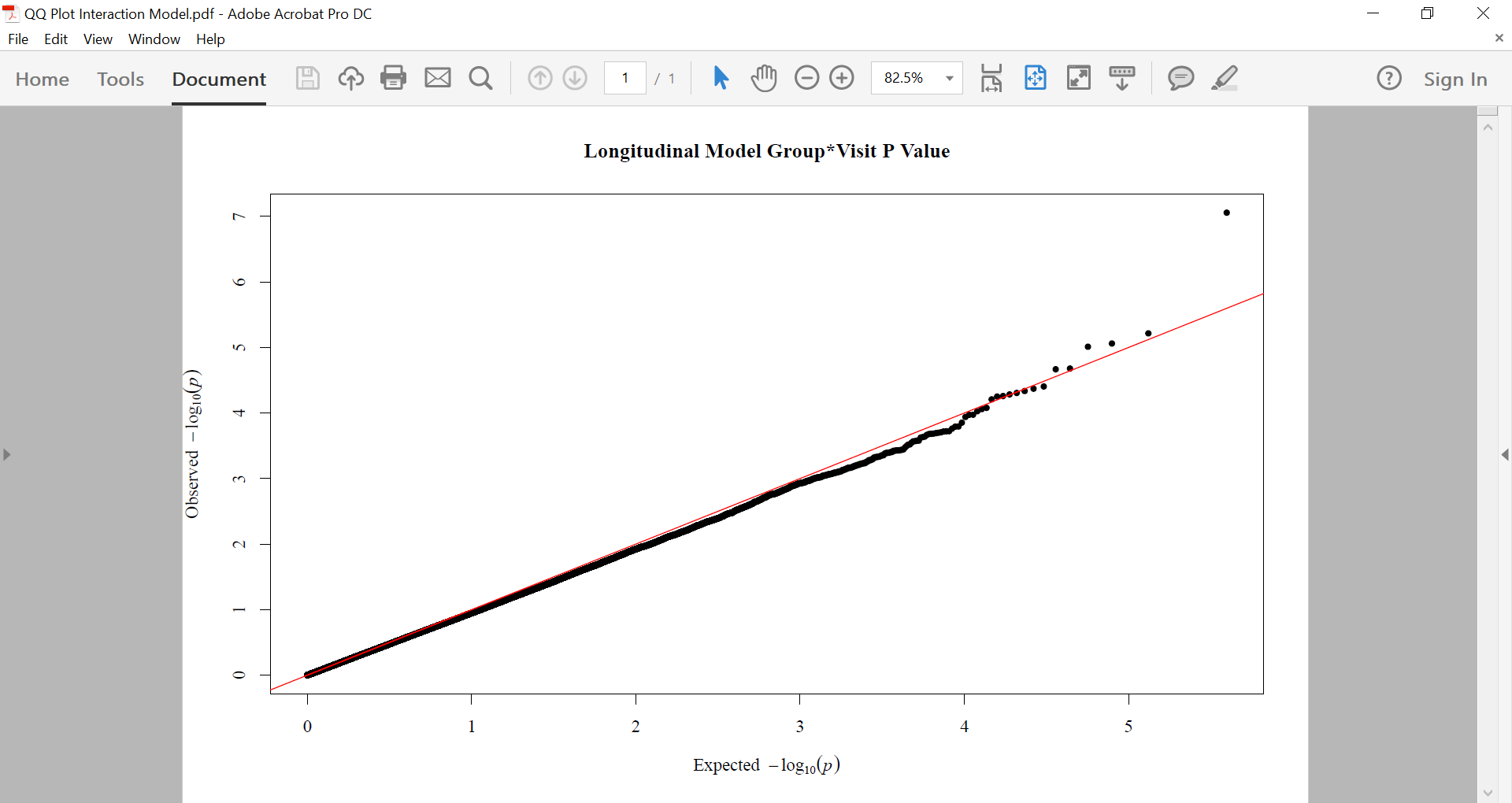


**Appendix Figure C.** Genomic inflation and model diagnostics for the group*visit longitudinal model**.** The interaction model tested the null hypothesis that the difference in methylation between the post-IA and pre-IA visits does not differ across the three autoimmunity phenotypes. Review of the Q-Q plot (**Appendix Figure C**) in combination with traditional lambda (0.926) and Bacon estimated lambda values (0.9350) suggested genomic inflation was not a concern in the longitudinal interaction model.

**Supplementary References:**

1. van Iterson M, van Zwet EW, Heijmans BT, Consortium B. Controlling bias and inflation in epigenome-and transcriptome-wide association studies using the empirical null distribution. *Genome biology.* 2017;18(1):19.
