# Supplementary material for: Longitudinal changes in DNA methylation during the onset of islet autoimmunity differentiate between reversion versus progression of islet autoimmunity
Source: Front Immunol. 2024 Jun 10;15:1345494. doi: 10.3389/fimmu.2024.1345494 (PMC11194352; doi:10.3389/fimmu.2024.1345494)
Supplement: Supplementary file 3 [file DataSheet_3.docx]

| **Appendix Table B.** Distribution of demographics and clinical characteristics in the overlapping populations | | | | | | | |
| --- | --- | --- | --- | --- | --- | --- | --- |
|  |  | **Full Population (n=143)** | | **Gene Expression Overlap (n=36)** | | **Metabolite Overlap**  **(n=110)** | |
|  | | **n \| mean** | **% \| stdev** | **n \| mean** | **% \| stdev** | **n \| mean** | **% \| stdev** |
| **Group, n (%)** | |  |  |  |  |  |  |
|  | Maintainer | 60 | 42.0% | 21 | 58.3% | 49 | 44.5% |
|  | Progressor | 42 | 29.4% | 6 | 16.7% | 27 | 24.5% |
|  | Reverter | 41 | 28.7% | 9 | 25.0% | 34 | 30.9% |
| **Non-Hispanic White, n (%)** | | 110 | 76.9% | 26 | 72.2% | 85 | 77.3% |
| **Female Sex, n (%)** | | 74 | 51.7% | 19 | 52.8% | 57 | 51.8% |
| **DR3/4 High Risk Genotype, n (%)** | | 45 | 31.5% | 13 | 36.1% | 33 | 30.0% |
| **First Degree Relative with T1D, n (%)** | | 82 | 57.3% | 21 | 58.3% | 63 | 57.3% |
| **Age Pre-IA Seroconversion, mean (stdev)** | | 5.3 | 4.2 | 8.1 | 5.0 | 5.9 | 4.3 |
| **Age Post-IA Seroconversion, mean (stdev)** | | 7.4 | 4.3 | 11.3 | 4.1 | 7.7 | 4.3 |
