# Supplementary material for: Longitudinal changes in DNA methylation during the onset of islet autoimmunity differentiate between reversion versus progression of islet autoimmunity
Source: Front Immunol. 2024 Jun 10;15:1345494. doi: 10.3389/fimmu.2024.1345494 (PMC11194352; doi:10.3389/fimmu.2024.1345494)
Supplement: Supplementary file 4 [file DataSheet_4.docx]

| **Appendix Table C.** Distribution of autoantibody subgroups, defined based on first appearing autoantibody, within maintainers, progressors, and reverters | | | | | | | | | |
| --- | --- | --- | --- | --- | --- | --- | --- | --- | --- |
|  |  | **Maintainer (n=60)** | | | **Progressor (n=42)** | | | **Reverter (n=41)** | |
|  |  | **Freq \| Mean** | **% \| Stdev** | **Freq \| Mean** | | **% \| Stdev** | **Freq \| Mean** | | **% \| Stdev** |
| **Single Autoantibody at Seroconversion Visit** | | |  |  | |  |  | |  |
|  | IAA | 14 | 23.3% | 8 | | 19.0% | 16 | | 39.0% |
|  | GADA | 29 | 48.3% | 12 | | 28.6% | 16 | | 39.0% |
|  | ZNT8 | 5 | 8.3% | 5 | | 11.9% | 6 | | 14.6% |
|  | IA-2A | 1 | 1.7% | 4 | | 9.5% | 3 | | 7.3% |
| **Multiple Autoantibodies Present at Seroconversion Visit** | | 11 | 18.3% | 13 | | 31.0% | 0 | | 0.0% |
