# Supplementary material for: Longitudinal changes in DNA methylation during the onset of islet autoimmunity differentiate between reversion versus progression of islet autoimmunity
Source: Front Immunol. 2024 Jun 10;15:1345494. doi: 10.3389/fimmu.2024.1345494 (PMC11194352; doi:10.3389/fimmu.2024.1345494)
Supplement: Supplementary file 5 [file DataSheet_5.docx]

**Appendix 3.** Metabolites correlated with changes in DNAm pre- versus post-IA seroconversion

| **Appendix Table D**. Known metabolites from the HILIC panel correlated with one or more of the DNAm regions | | | | | |
| --- | --- | --- | --- | --- | --- |
| **Name** | **InChi Key** | **Pubchem ID** | **Average Mz** | **Average Rt min** | **DMRs*** |
| PC (38:5) | ZAYXPDDGEIJGGW-VSDNDEBUSA-N | 24778948 | 808.5834 | 4.31 | ∆DMR 8 |
| *DMR(s) correlated with metabolite levels in the secondary metQTM analysis | | | | | |

| **Appendix Table E**. Annotated metabolites from the lipid panel correlated with one or more of the DNAm regions | | | | | | |
| --- | --- | --- | --- | --- | --- | --- |
| **Name** | **InChi Key** | **Pubchem ID** | **Mode** | **Average Mz** | **Average Rt min** | **DMRs*** |
| PC(P-18:1(9Z)/20:1(11Z)) | VXNFJZDUQIFCMO-WCXGAVHKSA-N | 53480813 | Positive | 798.6385 | 6.20 | ∆DMR 9 |
| PE (38:4) | ANRKEHNWXKCXDB-BHFWLYLHSA-N | 46891781 | Positive | 768.5577 | 5.72 | ∆DMR 5 |
| TG (47:0) | DVSBIXGBJRWEQB-UHFFFAOYNA-N |  | Positive | 810.7616 | 10.61 | ∆DMR 5 |
| TG (49:2) | QZYSUBAQYSVFNX-PSMULLBHSA-N | 9543993 | Positive | 839.7137/  834.7587 | 10.25/  10.21 | ∆DMR 2 and 5 |
| TG (49:3) | DIGMYZZFQSIQBD-PNLKURBTSA-N | 56938088 | Positive | 832.7432 | 9.84 | ∆DMR 8 |
| TG (51:3) | ISSGPXMQOMAFMJ-DMGKHJLRSA-N | 9544023 | Positive | 860.7733/  865.7284 | 10.30/  10.30 | ∆DMR 5 |
| TG (53:1) | WWJIBIGWKGFVAQ-JNSYTQKUSA-N | 9544081 | Positive | 892.8344 | 11.39 | ∆DMR 5 |
| TG (53:2) | RSINITWKVQRWSZ-RFVLVDBCSA-N | 9544102 | Positive | 890.8100/  895.7746 | 11.05/  11.05 | ∆DMR 5 |
| TG (53:3) | ZNQBEJJYVJSZLM-LEDQTTRKSA-N | 9544126 | Positive | 888.8054/  893.7603 | 10.69/  10.70 | ∆DMR 5 |
| LPC (15:0) | RJZVWDTYEWCUAR-JOCHJYFZSA-N | 24779458 | Positive | 482.3254 | 1.18 | ∆DMR 8 |
| PC (16:0/15:0) | NPGWXTIWUUFYAB-DIPNUNPCSA-N | 24778680 | Positive | 720.5601 | 5.05 | ∆DMR 8 |
| PC (15:0/18:1(11Z)) | GXTATYLPYLLNLV-MGIDVFSBSA-N | 24778662 | Negative | 804.575 | 5.14 | ∆DMR 5 and 8 |
| PC (33:2) | SBNDHGBVMZMSNL-UESLNCBNSA-N | 52922715 | Negative | 802.5594 | 4.68 | ∆DMR 8 |
| PC (35:1) | MFHIZGSSDZJFKD-IYEJTHTFSA-N | 52922679 | Negative | 832.6065 | 5.78 | ∆DMR 5 and 8 |
| PC (35:4) | LYNZMLHNPYEREG-ZHMZGHMTSA-N | 52922895 | Negative | 826.5595 | 4.59 | ∆DMR 8 |
| PC (36:3) | YPAZQMWFRMHBBM-CLKMJQEKSA-N | 53478785 | Negative | 842.592/  818.5485 | 5.12/  5.14 | ∆DMR 8 |
| PC(o-18:1(11Z)/16:0) | QCGUXAIDEOWPBV-SNKLRXETSA-N | 53481719 | Negative | 804.6115 | 5.84 | ∆DMR 8 |
| PC (33:0) | FHENRYRLCPXONH-LDLOPFEMSA-N | 52922645 | Positive | 748.5865 | 5.63 | ∆DMR 8 |
| PC (33:1) | OFRWLQSOUIZELQ-VEAYGOGPSA-N | 52922442 | Positive | 746.5719 | 5.19 | ∆DMR 5 and 8 |
| PC (33:2) | SBNDHGBVMZMSNL-UESLNCBNSA-N | 52922715 | Positive | 744.5563/  766.5422 | 4.72/  4.72 | ∆DMR 8 |
| PC (35:1) | MFHIZGSSDZJFKD-IYEJTHTFSA-N | 52922679 | Positive | 774.603 | 5.82 | ∆DMR 8 |
| PC (35:3) | AYXGHIQPMDYMJC-AHMBLZLYSA-N | 52924614 | Positive | 770.571 | 4.86 | ∆DMR 8 |
| PC (35:4) | OROZWUJCDDCYAU-IPUAOQJZSA-N | 52922204 | Positive | 768.5553 | 4.63 | ∆DMR 8 |
| GPCho (36:3) | WZCLAXMADUBPSG-RIXBAXMTSA-N | 52922657 | Positive | 784.5899/  806.5697 | 5.17/  5.14 | ∆DMR 8 |
| *DMR(s) correlated with metabolite levels in the secondary metQTM analysis | | | | | | |
